# Eco-Friendly Sustainable Nanocarriers to Treat Oxidative Stresses and Skin Aging-Related Ailments, Valorization of a By-Product

**DOI:** 10.3390/bioengineering10070798

**Published:** 2023-07-03

**Authors:** Zaheer Ullah Khan, Taous Khan, Hira Khan, Naveed Ullah Khan, Yang Ding, Atif Ali, Jiang Ni

**Affiliations:** 1 Department of Pharmacy, COMSATS University Islamabad, Abbottabad Campus, Abbottabad 22060, Pakistan; zaheerkhan2092@gmail.com (Z.U.K.); taouskhan@cuiatd.edu.pk (T.K.); 2 Department of Pharmacy, Abbottabad University of Science and Technology, Havelian, Abbottabad 22500, Pakistan; hirakhan@aust.edu.pk; 3 Department of Pharmacy, CECOS University of Engineering and Emerging Sciences, Peshawar 25000, Pakistan; naveedkhan1676@hotmail.com; 4 College of Pharmacy, Pharmaceutical Series, China Pharmaceutical University, Nanjing 210000, China; dydszyzf@163.com; 5 Department of Pharmacy, Affiliated Hospital of Jiangnan University, Wuxi 214000, China

**Keywords:** *Citrus sinensis* L. peels, NLCs, oxidative stress, skin aging, non-invasive skin investigations

## Abstract

The peel from *Citrus-sinensis* L. is a medicinally significant food waste, and its extract (O-Ext) could be significant against oxidative stresses and skin aging, However, the penetration barriers, instability in formulation, undefined toxicities, and enzymatic activities make the O-Ext difficult to formulate and commercialize. The goal of this study was to evaluate O-Ext against oxidative stress, prepare O-Ext-loaded nano-lipid carriers (O-NLCs), and load them into topical O/W-emulsion (O-NLC-E) to improve O-Ext permeation and its in vivo antiaging effects. TPC, TFC, DPPH activity, and mineral/metal contents of O-Ext were determined via atomic-absorption spectroscopy. For bioactive compounds profiling, GC-MS analysis was carried out. O-NLCs were prepared and tested for physicochemical attributes, while HaCaT and fibroblast cells were used to study permeation and cytotoxicity. The kinetic characteristics of ex vivo permeation through rat skin were established, following the Higuchi model. Following written consent, safety investigations were conducted on human volunteers for three months, where optimized O-NLC-E and B-NLC-E were regularly applied on cheeks. Non-invasive procedures were used to assess the volunteer’s skin erythema, TEWL, sebum level, melanin, hydration, pH, elasticity, and pore sizes after specified intervals. The results demonstrated that applying O-NLC-E formulation to the skin of volunteers directed significant antiaging benefits. The study offers nanotechnology-based sustainability approach against skin ageing.

## 1. Introduction

Skin aging is a natural process and major cause of many degenerative diseases. The utmost instinctual fallout of the aging is exteriorized on the skin, which can be debilitating for the structure, function, and appearance of skin. It is directly related to ugly appearance, increased number of wrinkles, laxity, emotional discomfort, elastosis, and telangiectasia. Various intrinsic and extrinsic factors can accelerate the skin aging by promoting oxidative stresses via enhancing the free radical concentration than normal. The overproduction of free radicals’ and oxidative stresses may be controlled via antioxidants and anti-oxidative enzymes such as superoxide dismutases (SOD), GST, GSH, POD, and catalase. Enriched extracts from natural compounds demonstrate good antiaging activity by reducing free radicles and oxidative stress, and mostly exhibit high safety profile. Due to excellent biosafety, natural compounds based pharmacotherapeutics are in large demand, and this demand is increasing day-by-day throughout the globe [1,2]. Natural bioactive compounds can be found in the food wastes, also called biowastes, and their effectual use for human health will be an excellent sustainability approach. It has been reported that guava, grapes, and citrus fruits produce 10, 20, and 30–50% of waste, respectively, compared to their total mass [3]. The utilization of these biowastes’ extraction as antiaging agents can lessen the environmental load, as well as the stress on medicinal plants, which are already 100-fold greater than the extinction rate [4]. It is a great alternative to costly imported natural antiaging agents and a step towards the use of sustainable and green resources.

Orange (*Citrus sinensis* L.) peel is a major industrial waste [5] containing many flavonoids, such as hesperetin, hesperidin, neodiosmin, nobiletin, kaempferide, naringenin, vanillin, rutin, and other phenolic acids, such as chrologenic, caffeic, ferulic, cinnamic, ascorbic acid, vanillic, and p-coumaric [6,7]. Moreover, orange peels extract (OPE) shows promise within the dermatology arena [5] as an antioxidant, as well as demonstrating anti-carcinogenic, anti-inflammatory, and anti-aging agents [7,8]. It has been also reported to demonstrate tyrosinase inhibitory activity, which regulates melanin formation in the skin and, thus, could be used as skin whitening agent [6,9]. In conventional topical formulations, OPE have some limitations such as low permeation across stratum corneum, enzymatic degradation, and lower chemical stability [10].

Nanotechnology is an emerging drug delivery tool and can be effectually utilized for the delivery of natural bioactive compounds. Nanostructured lipid carriers (NLCs) represent an innovative delivery system made up of lipids materials, surfactants, and cosurfactants that may be administered topically, dermally, or trans-dermally. In topical applications they have some unique properties such as increased skin occlusion, increased skin hydration and elasticity, improved skin permeation, drug targeting, improved benefit/risk ratio, improved UV blocking activity, and improved chemical stability of chemically labile compounds [11,12]. The formulation components are safe and fall under the Food and Drug Administration’s “generally recognized as safe” (GRAS) [13]. The use of green surfactants in the preparation of NLC containing extract converts them into green formulations with fewer side effects and low cost. The NLCs formulations with green surfactants can be the most suitable delivery system for bioactive compounds and will be an impactful sustainability approach.

**Figure 1:**
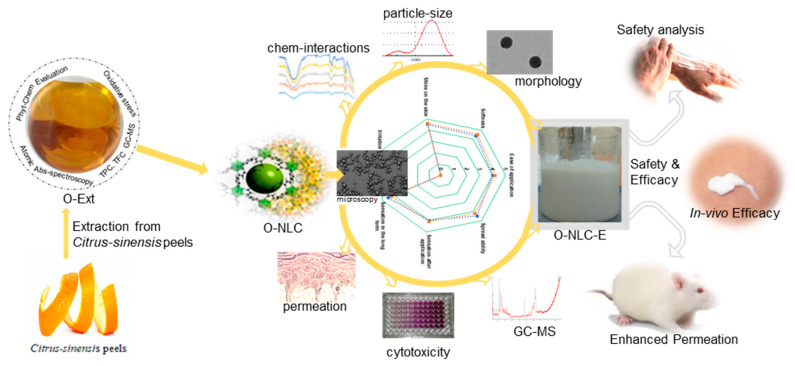
*Citrus sinensis* peels extract was evaluated for phytochemicals (GC-MS, TPC, TFC). The extract was then loaded into nanocarriers and delineated for in vitro and ex vivo evaluations. The in vivo efficacy was confirmed via human volunteers for non-invasive skin anti-aging parameters.

In this study, a sustainability approach was introduced, where orange peels extract was evaluated for antiaging activity and heavy metals concentration (safety). They were then loaded into NLCs (O-NLC) based on green surfactants (rhamnolipids) and characterized before loading into a secondary emulsion-based formulation (topical O/W emulsion) (O-NLC-E) as explained in Figure 1. For further assessments, physico-chemical characterization of O-NLC-E was performed and their ex vivo permeation was analyzed. For the in vivo evaluation, human volunteers were considered after their written consent were given and safety studies were conducted. The results showed a good safety profile and high antiaging efficacy. This sustainability approach was introduced; ① for the shelter of substantial bioactive compounds when applied to skin, ② to overcome the penetration barriers and improve the bioavailability of O-Ext, ③ to avert the enzymatic interaction, ④ to keep smooth antioxidant activities against oxidative stresses, ⑤ to improve economic and social acquiescence, and ⑥ providing the topical treatment with ease to fend off oxidative skin problems and early skin aging. Here, we offered a unique sustainability approach to defend the skin aging with naturally existed potential and superb biosafety.

## 2. Materials and Methods

### 2.1. Materials

Dimethyl-sulfoxide (DMSO), H_2_O, HCl, NaOH, DPPH, folin-ciocalteu reagent gallic acid (GA), AlCl_3_, ascorbic acid (AA), FeCl_3_, sodium phosphate, C2H_3_O_2_K, HNO_3_, HClO_4_, phosphate buffer, Cetyl alcohol, methanol, and Rhamnolipid 90 (Rh-90) were purchased from Sigma-Aldrich, while Folin–Ciocalteu reagent (FCR) and Na_2_CO_3_ were acquired from Merck (Dam-stadt, Germany). Analytical-grade pyragallol, dTNB, H_2_O_2_, EDTA, Hank’s balanced salt solution (HBSS), lipopolysaccharide (LPS), and cDNB were purchased.

### 2.2. Methods

#### 2.2.1. Preparation of *Citrus sinensus* L. Peels Extract

Orange peels were collected and shade dried after washing by distilled water; then, dry peels were ground into a fine powder which was then extracted using methanol (10:1, *v*/*w*) via stirring. Extract was filtered out and concerted for drying via a rotary-evaporator. The percentage yield was calculated via Equation (1).

(1) $$WO\;\left(\%\right)=\frac{WOE}{WOP}\times 100$$

where *WO*% is the percent yield of orange peels extract (O-Ext), *WOP* is the weight of orange peels powder, and *WOE* is the weight of dry extract.

#### 2.2.2. Total Phenolic Contents (TPC)

The TPC of O-Ext was ascertained via the FCR [14,15]. A volume of 110 μL of extract and FCR was shifted into the 96-wells plate and at 37 °C incubated for 5 min. Then, Na_2_CO_3_ (7% *w*/*v*) was added for a final volume of 200 μL. Then, the mixture incubated readings were taken at 765 nm (Biotech USA, Hungry, microplate reader Elx 800). The TPC were stated as milligrams of gallic acid (standard) equivalents per gram of dry O-Ext [14].

#### 2.2.3. Total Flavonoid Contents (TFC)

The TFC of O-Ext was ascertained via the calorimetric method [14,15]. An amount of 5 mg of O-Ext was dissolved in 5 mL of methanol to prepare stock solution. Next, 40 μL of O-Ext, 10% aluminium chloride, and 1M C_2_H_3_O_2_K solution were shifted into a 96-well microplate, followed by the addition of 160 μL of distilled water to bring the finalvolume up to 0.2 mL, which was then incubated for 30 min. A microplate reader was used to measure absorbance at 415 nm (Biotech USA, Elx 800). The TFC were expressed as milligrams of quercetin equivalent per gram of dry O-Ext [14].

#### 2.2.4. Antioxidant Assay

The O-Ext antioxidant capacity was determined using the DPPH-assay [16]. Then, 100 mM solution of DPPH (10% DMSO) was prepared and 90 μL of the solution was mixed with O-Ext in a 96-well plate and at 37 degrees Celsius and incubated for 0.5 h [17]. The absorbance drop was assessed at 517 nm using a microplate reader (Synergy HT BioTek^®^ USA, Hungry). Ascorbic acid (AA) was used as a standard. Percent inhibition was calculated by Equation (2).

(2) $$\;Inhibition\;\left(\%\right)=\frac{\left(Abs\;of-ve\;control)-(Abs\;of+ve\;control\right)}{\left(Abs\;of-ve\;control\right)}\times 100$$

#### 2.2.5. Atomic Absorption Spectroscopy

The extract was analyzed via Atomic Absorption Spectrometer AAnalyst (PerkinElmer, Waltham, MA, USA) for different elements examination. 500 mg of O-Ext was added to a conc. HNO_3_ and HCL mixture (3:1). The mixture was heated up to 85 °C and 1 mL of HCLO_4_ added. Then, distilled H_2_O was mixed to attain a final volume of 50 mL, which was injected into the sample for analysis [14,18].

#### 2.2.6. Identification of Compounds in O-Ext via GC-MS

Utilizing GC-MS analysis, compounds in the O-Ext were analyzed and screened. The GC-MS experiment was carried out according to protocol [14]. Identification was confirmed using calculated fragments, molecular mass, and molecular structure. Mass-spectrum analysis was performed using a database from the NIST-library, containing more than 62,000 molecular patterns [14].

#### 2.2.7. Effect of Extract on Oxidative Stress; Superoxide Dismutases (SOD), GST, GSH, POD, and Catalase

Peritoneal macrophages of mouse were activated with LPS to examine the O-Ext effect on oxidative-stress indicators such as GST, GSH, SOD, POD and catalase, as published previously [14]. GSH was calculated by adding the cDNB as previously published, and quantification was carried out at 340 nm. While using dTNB, the GST was quantified 350 nm. Catalase was analyzed at 240 using H_2_O_2_ buffer. The SOD concentration was assessed by mixing Tris-EDTA (50 mM and pH 8.5) and pyragallol (24 mM), and the absorbance was noted at 420 nm. The SOD contents were determined using pyragallol (24 mM) in 50 mM tris-EDTA buffer (pH 8.5) at 420 nm. The POD contents were calculated as previously published [19].

#### 2.2.8. Preparation of NLCs

##### Selection of Solid Lipid and Liquid Lipid

The O-Ext solubility in different solid lipids (cetyl alcohol, precirol ATO-5, Compritol888ATO, Glycerin monostearate, bee wax and stearic acid) was determined by dissolving it, in ascending order in defined quantity with stirring (100 rpm) till saturation after 24 h of stirring at 85 °C. In the case of oils (olive oil, oleic acid, MCT, sesame oil, avocado oil and black seed oil), the solubility was determined and oil with high solubility were assigned for further process [20].

##### Solid Lipid and Oils Compatibility

To analyze the compatibility of the selected solid lipids (SL) and oils, a screening test was carried out. SL were melted and mixed with oils at high temperature and then cooled down in a vial with horizontal rotation to form a film on the wall of vial. The crystals formation due to the incompatibility and phase-separation (PS) were observed [21].

##### Surfactant and Cosurfactant Selection

To select most suitable surfactant, 300 mg of tween20, tween80, Pluronic F68, Poloxamer188, and rhamnolipids 90 were added to the lipid mixture (300 mg) consisting of 85% solid lipids and 15% oil, and gently heated. Then, 50 mg of the mixture was diluted via H_2_Od up to 50 mL; the stability of the prepared emulsion was observed up to 2 h. The transmittance (clarity) was observed at 638 nm and surfactant giving high transmittance was used for further experiment [22,23]. Soya lecithin was selected as a cosurfactant as reported previously in our lab [24].

##### Development of NLCs

The high shear rate homogenization approach was employed to generate NLCs as described previously [14]. The organic phase (OP) was formed by fully dissolving lipids mixture, soya lecithin in ethanol, and extract. The OP was heated and added drop by drop to the aqueous-phase (AP) containing surfactant during homogenization at 10 k rpm. The resulting dispersion, containing O-Ext loaded NLCs (O-NLCs), was sonicated and cooled to room temperature. The dispersion was lyophilized to obtain pure O-NLCs. Unloaded NLCs (B-NLCs) were synthesized without extract [10,25]. To avert damage, 3.0 percent mannitol was used as a lyoprotectant [26].

##### Optimization of NLCs

To obtain optimized NLCs, the Box–Behnken model was used; 15 Formulations were produced to find out the effect of independent factors (amount of lipids mixture, solid lipid (cetyl alcohol) amount in lipids mixture, surfactant amount) on dependent factors (Size (nm), zetapotential (mV) and polydispersity index) as shown in Table S2. The composition of produced formulations and their results are shown in Table S1. Suggested optimized formulation with predicted and actual results are shown in Table S3.

#### 2.2.9. O-NLCs Characterization

##### Zetapotential, Size and Polydispersity Index (PDI)

B-NLCs and O-NLCs were diluted with H_2_Odd (X20). The physicochemical properties were found out via Nano-ZS90 (Malvern Instruments Ltd., Malvern, UK) at 25 °C [26].

##### Entrapped Phenolic Contents

Free or unentrapped amount of O-Ext was determined in O-NLCs dispersion via centrifugation (5000 rpm) of dispersion in 15 mL MILLIPORE tube of 30,000 MWCO. The *PC* was determined in collected solution at 765 nm using UV visible spectroscopy [27]. The percent EE was calculated using the given Equation (3):

(3) $$EP\;\left(\%\right)=\frac{{PC}_{total}-{PC}_{free}}{{PC}_{total}}\times 100$$

where, *PC_total_* = total amount of phenolic compounds, *PC_free_* = amount of lipids.

##### Fourier Transmission Infra-Red Spectroscopy

To confirm the entrapment of O-Ext in O-NLCs and the interaction between B-NLCs components and extract, FTIR-spectroscopy (Tensor 27, Bruker, Nijmegen, The Netherlands) was performed [26,28].

##### Morphological Observation

Transmission-electron-microscopy (JEM-1200EX, Jeol, Groningen, The Netherland) was utilized to examine the morphology of O-NLCs [29].

##### Cytotoxicity Studies via MTT Assay

For cytotoxicity experiments, human-keratinocytes (HaCaT) and fibroblast cells were utilized [14]. These were grown in DMEM and RPMI 1640, respectively. In incubation, both cultures were supplemented with FBS, streptomycin, and penicillin [30]. Following incubation, the medium was sucked out, and replenished with new medium. Test samples were added and incubated for the appropriate duration. The MTT solution was added before incubation and gently shaken for 15 min. The absorbance was measured at 570 nm, and viability of cells was computed using Equation (4) [31].

(4) $$Cell\;viability\;\left(\%\right)=\frac{OD\;values\;of\;dosing\;group-blank}{Control\;group-blank}\times 100$$

##### Cells Permeation Studies

HaCaT cells were cultivated into a 24-well Transwell insert at a concentration of 105/cm^2^ to discover O-NLCs’ permeation, as described in our previous publications [14,24]. Later, between 17 and 21 days, the transepithelial-electrical-resistance was assessed using an EVOM-ohmmeter (Sarasota, FL, USA) to examine the stability of the cells. Cells were washed off three times before adding 60 μg of O-Ext-E (Emulsion containing O-Ext), and 0.2 mL HBSS (300 μL/mL) O-NLCs to the top of the Transwell and 1.0 mL HBBS to the lower chamber. Cells were then kept at 37 degrees with horizontal shaking at 50 to 60 rpm per min. A volume of 500 μL of HBSS was collected at various intervals (1–6 h), and equivalent quantity of HBSS was replenished in the inferior chamber. The materials were freeze-dried. As previously stated, the *PC* was evaluated [27]. The carriage mass (Q) was computed via Equation (5):

(5) Qm=Ci×V1+∑Ci−1×0.5

where, “*Qm*” signifies the carriage transport-mass (µg); “*V*1” is the mass of solution in inferior-chamber; and *Ci* is the *PC* (µg/mL) [24,32].

#### 2.2.10. Preparation of O-NLCs Loaded O/W Emulsion

An emulsion is a colloidal system of a minimum of two non-miscible liquids where one is dispersed in another one [33]. To prepare the o/w emulsion, water phase was heated on a hot plate above 70 °C, containing sodium behenoyl lactylate 3.00% (emulsifier), vegetable glycerin 5.00% (humectant), acacia senegal gum 2.00% (polymer), sodium benzoate 1.00% (preservative), glyceryl stearate 2.00% (emulsifier), and liquid paraffin 5.00% (emollient and moisturizer). Then, oil phase was added drop wise while stirring; 2.0% silica was added as a sensory modifier. The system was then homogenized by high shear homogenizer (Ultra Turax) for 10 min at 15,000 rpm. During homogenization, O-NLCs and O-Ext (maintaining 2% O-Ext concentration) were added to produce O-NLCs loaded emulsion (O-NLC-E) and O-Ext loaded emulsion (O-E); while in production of B-NLCs loaded emulsion, B-NLC-E unloaded NLCs (B-NLCs) were added. At the end, the homogenizer speed gradually reduced to avoid the formation of bubbles, and the temperature of the colloidal system was reduced to room temperature [34].

##### Characterization of O-NLCs-E

- Dilution test and microscopic evaluation

Emulsion was diluted with distilled water to confirm the emulsion type. The microscopic studies of diluted O-NLC-E were performed using optika microscope and images were taken with an attached camera [35].

##### Stability Studies

- Accelerated Thermodynamic stability studies

Accelerated thermodynamic stability tests are known worldwide as suitable for guessing the shelf-life of formulations [36]. In thermodynamic stability studies, 3 cycles of centrifugation (C) at 5000× *g* rpm for 5 min and heating–cooling (HC) cycle, and 4 °C for 48 h, followed by 45 °C for 48 h, were conducted. In freeze–thaw (FT) cycles, the formulation was kept [37].

##### pH

The pH is regarded as an eminence criterion for topical formulations, especially emulsions [38]. The pH of O-NLC-E was assessed directly with a pH-meter (HANNA EDGEPH, HANNA, Padua, Italy). Readings were taken in triplicate over a 90-day period to assess pH fluctuation at various storage temperatures, namely, 8, 25, 40, and 40 °C + 75Rh [27].

##### Chemical Stability

Chemical stabilities of O-NLC-E were studied via the finding of *PC*, comprising its chief compounds by UV spectroscopy. *PC* (%) drop after storing was estimated via Equation (6):

(6) $$Percentage\;reduction\;\left(\%\right)=\frac{Mpc-Mo}{Mo}\times 100$$

where *Mpc* is *PC* amount of afterward storage (mg/L) and *Mo* before storage (mg/L) [37].

##### Ex Vivo Diffusion Studies

For ex vivo diffusion analysis of O-E and O-NLC loaded emulsions, the 11 mL of PBS (pH 5.5) was filled in a receptor-compartment of Franz’s diffusion cell and 1 g testing emulsion in a donor-compartment. To avoid evaporation, the formulation was wrapped with parafilm. The PBS in the receptor-compartment was stirred at 37 °C at 210 rpm. At 0.5, 1, 2, 4, 8, 16, 24, 36, and 48 h, 1.0 mL of sample was replaced by fresh PBS for analysis. To determine the phenolic contents of the samples, a Shimadzu 1800 UV-visible spectrophotometer was utilized at 765 nm [27].

##### Rheological Studies of O-NLCs-E

The viscosity of the formulation was measured with a rheometer (RM200 TOUCH, LAMY RHEOLOGY, Champagne-au-Mont-d’Or, France) and Rheomatic-P software. A spindle R-III measuring-system twelve was employed. The viscosity was evaluated at 25 ± 5 °C for 60 s at a speed of 100 rpm. The measurements were as follows: Viscosity = f (time). The viscosity = f (Time) equation enables for measuring at a stationary shear rate over a specified time period [39]. The following measurement protocols were used: pre-shearing time of 5 s, pre-shearing rate of 10 s, time of 60 s, shear rate of 50 s, and an immediate start.

##### Non-Invasive Skin Investigation of O-NLCs-E

- Safety evaluation of O-NLCs-E in human volunteers

Patch testing was used to perform safety testing (B-NLC-E versus O-NLC-E) on the forearms of 11 participants under ethical committee approval CIIT/ATD/BSC/17-07 [40]. An area of 5 cm × 4 cm on both forearms was blotted. Baseline erythema levels were determined using a probe fitted to the Multi-Skin-Test Center MC 1000 (Kazak, Gebraucht, Germany). Self-application by B-NLC-E and O-NLC-E volunteers was used. Each was applied to the blotting regions on each forearm in separate tests (B-NLC-E versus O-NLC-E). Following treatment of the B-NLC-E and O-NLC-E, the areas were covered with surgical dressing. After 48 h, the dressings were removed and the amount of erythema on both forearms was measured again [41,42,43].

##### Effect of O-NLCs-E on Different Skin Aging Parameters via Long Term Use

Face studies were conducted using single-blinded non-invasive procedures, with a three-month of recommendation. The project procedures were permitted by the ethical committee of the Institution under the code CIIT/ATD/BSC/17-07. After receiving written agreement, preparations for left and right cheeks were given to the 11 volunteers for consistent usage until the end of the research period. Complete Skin Investigation (CSI) was utilized to examine skin melanin level, erythema, TEWL, moisture contents, sebum level, and wrinkles [44,45].

##### Panel Test of O-NLCs-E

Volunteers evaluated the sensory characteristics of the formulations. They were instructed to apply a standardized amount, 200 mg/cm^2^, of the formulations and answered a sensory questionnaire with values from −5 to +5 representing very harmful to very beneficial, respectively, to evaluate sense of softness, spreadability, sense in long-term, sense just subsequently application, easiness of application, shine, and irritation of the formulations [46,47].

### 2.3. Statistical Analysis

All assays were in triplicates (*n* = 3), and statistical analysis was carried out using *t*-test and two-way ANOVA (*p* < 0.05) using excel and GraphPad prism v8 software.

## 3. Results

### 3.1. Preparation and Evaluation of O-Extract

The fine powder of orange dried peels methanolic extraction (O-Ext), concentrated via rotary evaporator giving 24.7% yield, was obtained. The MeOH was selected for extraction as it has amphoteric-nature, due to which it is able to extract maximum bioactive compounds. The yield was compared with the reported study conducted by Liew et al. on *Citrus sinensis* peels [48]. The procedure was revised thrice to obtain maximum extraction [14].

#### 3.1.1. Total Phenolic and Flavonoids Contents

TPC of O-Ext was 170.5 mg GAE/g of dry-weight. Phenolic compounds are secondary metabolites and have been used to treat premature skin aging [49] because they act as inhibitors of pro-inflammatory-mediators’ (PIMs), which induce “inflame-aging” [50]. The flavonoids content in O-Ext were determined via a calorimetric method, and reported 31.5 mg QE/g as dry weight of O-Ext. Flavonoids assist in maintaining mitochondrial function, postponing aging, and extending healthier life [51]. They can condense lipo-protein damages on cytomembrane, and modify numerous signaling-pathways, e.g., inhibiting xanthine-oxidase a cause of oxidative-stress which accelerates skin aging [52].

#### 3.1.2. Antioxidant Assay

The IC50 of O-Ext found via DPPH assay was 24.70 μg/mL, and for ascorbic acid (AA) was 5.50 μg/mL. Free radical scavenging activity has strong association with phenolic contents, and the high value of DPPH indicates the potency of extract towards scavenging of free radicals [53]. Free radicals’ scavenging property decrease damaging during oxidative stress and makes it suitable as an antiaging agent to use in cosmeceutical and pharmaceutical formulations [54,55].

#### 3.1.3. Atomic Absorption Spectroscopy

To create a suitable antiaging natural-based sustainable delivery system, metals assessment is essential for O-Ext [56]. For this purpose, analysis of the heavy metals, i.e., As, Cr, Co, Cd, Fe, and Pb, was carried out and results are shown in Table 1. It was noticed that levels of heavy metals were lower than permitted ranges [14]. In O-Ext, the “As” was undetectable and under EU, Canada, and Germany laws, topical medication delivery cannot use any of its salts [57]. It is causative agent of cancer, hair loss, and age spots [58]. In O-Ext, Cr was present at 0.032 ppm, and according to US-FDA, a maximum of 50.0 ppm concentration is allowed [59]. Co was found to be present at 0.083 ppm in O-Ext, as is a part of important vitamins, e.g., B12, but repeated contact could cause contact intoxication, which leads to irritation and rashes [60]. The level of lead in O-Ext was 0.897 ppm. Lead as a contaminant in cosmetology is permitted at a maximum amount of 20.0 ppm, according to the US-FDA. O-Ext had a Cd of 0.028 ppm and is allowed in pharmaceutical preparations from 0.03 to 0.10 ppm; it is utilized as a coloring agent in formulations [61]. Fe was found at 0.332 ppm, which is within a suitable range [61,62]. According to the statistics, which are in line with US-FDA reports and guidelines, O-Ext is appropriate to use in topical preparations as an antiaging agent.

#### 3.1.4. Identification of Compounds in O-Ext via GC-MS

**Figure 2:**
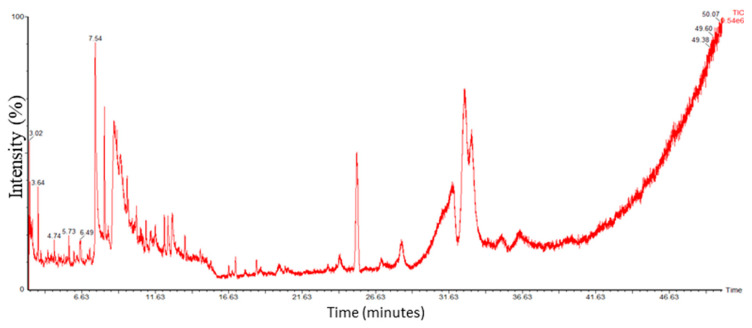
GC-MS Chromatogram of O-Ext.

The bioactive compounds in O-Ext were identified via GC-MS (Figure 2). The molecular weight (M.W), molecular formula (M.F), name, and CAS number of compounds were found using NIST library given in Supporting Table S1. The O-Ext showed different compounds such as Dodecanoic acid; Linalool; Alpha.-terpineol; Hentriacontane; Cyclohexasiloxane, dodecamethyl; Heptadecane, 1-bromo; L-norvaline, n-(2-methoxyethoxycarbonyl); 2,4-di-tert-butylphenol; 2,6-octadien-1-ol, 3,7-dimethyl-, propan; 5,5-dimethyl-cyclohex-3-en-1-ol; Benzoic acid, 2-ethylhexyl ester; Methyl 2-hydroxy-pentacosanoate; Methyl 11-methyl-dodecanoate; Tetradecanoic acid, 10,13-dimethyl-, met; Ethyl 14-methyl-hexadecanoate; N-hexadecanoic acid; Oleyl oleate; Isopropyl linoleate; Hentriacontane; and 2,6,11-dodecatrienal, 2,6-dimethyl-10-me. Mostly, detected compounds given in Supporting Table S1 are reported with good antioxidant and antiaging activities, mostly used in topical anti-aging and skin care formulations.

#### 3.1.5. Effect on Antioxidant Enzymes

**Figure 3:**
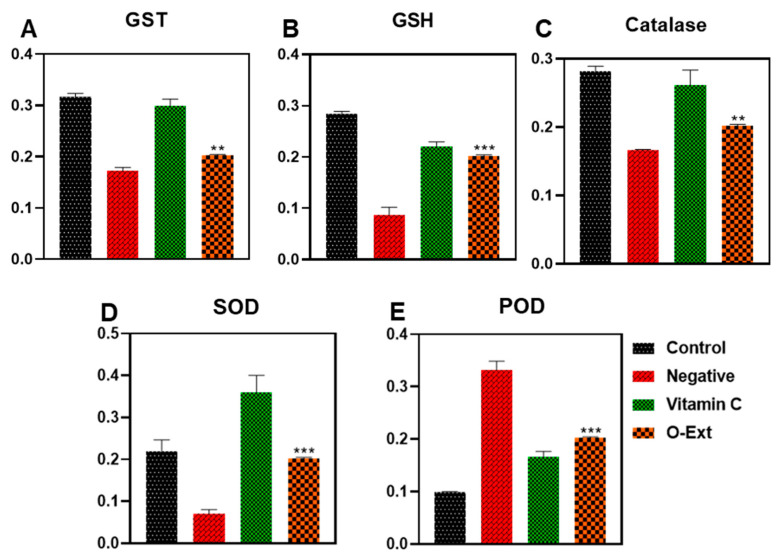
Effect of O-Ext on antioxidant enzymes using macrophages (**A**) GST, (**B**) GSH, (**C**) Catalase, (**D**) SOD, (**E**) Peroxidase. Data are presented as mean ± SD (n = 3). Where ** *p* < 0.01, *** *p* < 0.001.

Oxidative stress is the cause of many pathological conditions and disturbances, where antioxidant enzymes play a homeostatic role. It speeds up the natural aging process. In human dermis, different external and internal factors intervene and lead to overproduction of FRs which leads to the oxidative stress and suppression of AOE, resulting in premature skin aging involving in skin hyperpigmentation, sagging, wrinkling, roughness, and dryness. In the present study, the peritoneal macrophages of mouse were used to evaluate the O-Ext effect on antioxidant enzymes such as GST, GSH, catalase, POD, and SOD [14]. The GSTs are called for biosynthesis and transport of endogenous molecules and cell defense by catalyzing reduced glutathione conjugation through the cysteine thiol [5]. The O-Ext noticeably made the glutathione-s-transferase (GST) concentration comparatively to the negative control, as demonstrated in Figure 3A. Glutathione (GSH) plays a serious part in redox homeostasis sustaining [6]. O-Ext raised the GSH concentration compared to the negative control group, Figure 3B. GSH in cytosol (1–10 mM) plays a major role in removal of ROS which are catalyzed by GSTs [6]. Catalase breaks down H_2_O_2_ to H_2_O and O_2_, lowering the oxidative stress inside cells [7]. The extract displayed a significant surge compared to the negative control in the catalase analysis, Figure 3C. In Figure 3D, the SOD results that are presented indicate that compared to the negative control, the O-Ext significantly raises the concentration of SODs. It catalyzes the transformation of superoxide (O_2_) into less hazardous H_2_O_2_ [7]. Protein carbonylation, DNA damage, and membrane lipid-peroxidation are all associated with decreased SOD activity. A well-known method used to detect oxidative stress in cells is lipid peroxidation (POD). In the LPS-treated negative control group, the POD content was significantly higher, as seen in Figure 3E; the O-Ext significantly reduced the amount of the POD (GPx) in comparison to the LPS-treated group. The quantity of POD was, likewise, significantly lowered by the positive control. Overall results given in Figure 3 reveal that O-Ext could lower oxidative stress, which is why it is a suitable natural agent against aging. It could be used in topical formulation as a natural antiaging agent.

### 3.2. Fabrication and Evaluation of O-Ext Loaded NLCs

#### 3.2.1. Components Selection and Their Compatibility for NLCs Preparation

Among the solid lipids based on solubility cetyl alcohol, beeswax and stearic acid were selected, and olive oil, oleic acid, and avocado oils were selected based on solubility for further compatibility tests. The cetyl alcohol and oleic acid combination showed more compatibility than other mixtures, as shown in Figure S1. For selection of a suitable ratio of cetyl alcohol and oleic acid, they were mixed in different ratios (70:30 to 99.9:0.1), with the most suitable ratio having solid lipids ~90%, which was selected for further evaluation. In surfactant screening, the Rh 90 gives most clear emulsion with minimum inversion and high intensity.

#### 3.2.2. Optimization of NLC

**Figure 4:**
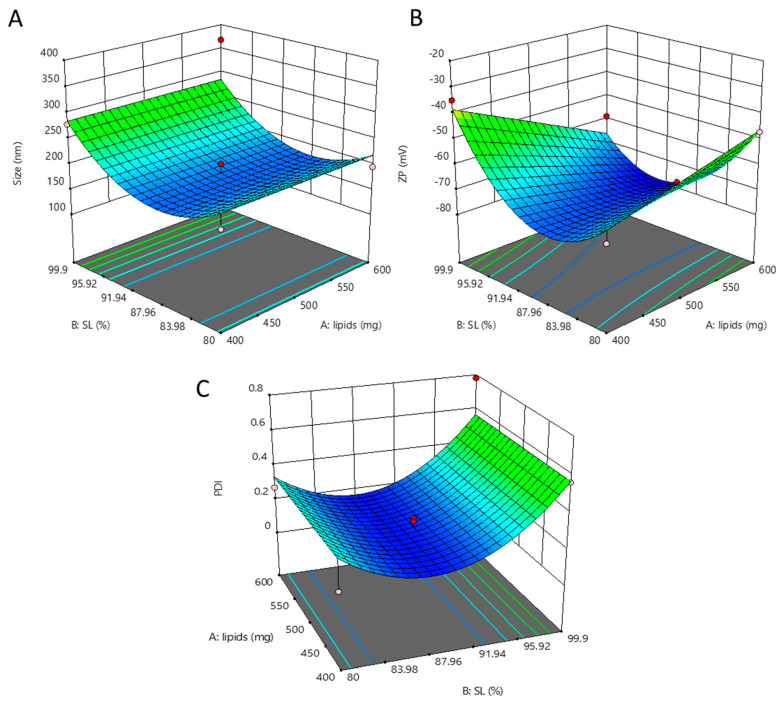
Effect of dependent factor on NLCs. Effect of solid lipid (B: SL) amount in lipid mixture and total lipid mixture amount (A: lipids) in optimized formulation keeping surfactant concentration constant (**A**). Effect of solid lipid (B: SL) amount in lipid mixture and total lipid mixture amount (A: lipids) in optimized formulation keeping surfactant concentration constant (**B**). Effect of solid lipid (B: SL) amount in lipid mixture and total lipid mixture amount (A: lipids) in optimized formulation keeping surfactant concentration constant (**C**).

The effect of B: solid lipid (SL) and A: lipid mixture maintaining constant C: surfactant amount is shown in Figure 4A–C. The graph shows that increasing and decreasing the SL (B) from 90% has a negative effect, i.e., increasing the size and selected range 600 mg lipid mixture (A) gives lower size then 400 mg lipids mixture. The effect of B: solid lipid (SL) and A: lipid mixture maintaining constant C: surfactant amount is shown in Figure 3. The graph shows minimum zetapotential at 600 mg lipids (A) and 90% (SL), while maximum potential (negative effect) was shown at 99.9% solid lipids. The effect of solid lipid (B: SL) amount in lipid mixture and total lipid mixture amount (A: lipids) in an optimized formulation maintains the constant surfactant concentration.

#### 3.2.3. Physico-Chemical Properties

**Figure 5:**
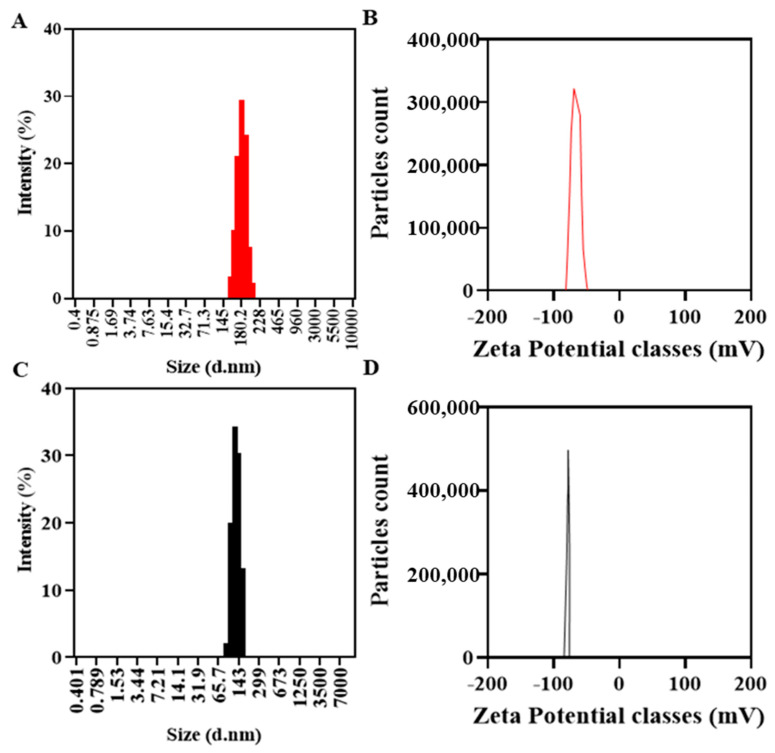
Physicochemical characterization, B-NLC size (**C**) and zeta-potential (**D**), and O-NLCs size (**A**) and zeta-potential (**B**).

O-NLCs demonstrated a size of 185.6 ± 13.5 nm, Figure 5A, 0.21 PDI, and 92.4 ± 1.8% EE. B-NLCs demonstrated a size of 164.5 ± 8.6 nm (Figure 5B) with 0.3 PDI, signifying homogeneity. The rise in O-NLCs PS was observed, and may have occurred due to the loading of O-Ext. The size distribution of O-NLCs was also augmented from 0.12 to 0.21. The PDI shows O-NLCs uniformity [63]. Yichao et al. (2020) reported the blank nanoparticles based on rhamnolipids with (118.7 nm) quite consistent with B-NLCs reported in this study [64]. Long Ba et al. (2016) also produced a nanoemulsion with same surfactant of about 130 nm [65]. The surface charge of unloaded NLCs (B-NLCs) was −77.70 ± 5.57 mV (Figure 4D). Rhamnolipids produce negative surface charge [64]. The high surface charge of NLCs is due to the RH, ascribed to its carboxylic groups [66,67]. Long Ba et al. (2016) described nanoemulsion (Rh-based) having −78.0 mV surface charge [65]. The O-NLCs showed −61.4 ± 6.18 mV (Figure 4C) surface charge, i.e., a small reduction in surface charge has occurred. The formulated O-NLCs bear the surface charge required for stable NLCs, i.e., <−30.0 mV, which is essential for stability of NP against aggregates formation [26]. The % EE of *PC* was 92.4 ± 1.8%. Park et al. (2018) reported turmeric nanostructured lipid carriers with 282 ± 7.19 nm diameter, 22.75 ± 1.20 mV zeta potential (-), and 93.3 ± 0.01% encapsulation efficiency [68]. 

#### 3.2.4. Fourier Transmission Infra-Red (FTIR) Spectroscopy

**Figure 6:**
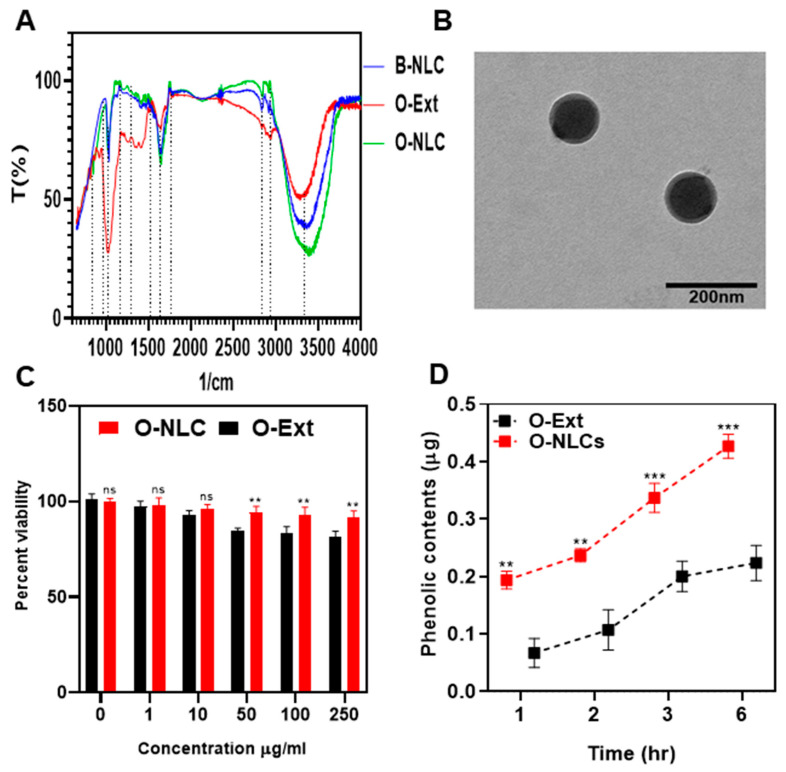
FTIR spectra of B-NLC, O-Ext and O-NLC (**A**), TEM of O-NLC (**B**), Cytotoxicity of O-Ext and O-NLC (**C**), and permeation of O-Ext and O-NLC across HaCaT cells (**D**). Data are presented as mean ± SD. n = 3. Where ** *p* < 0.01, *** *p* < 0.001, and ns = no significance.

FTIR analysis is a practical approach for quickly identifying loaded extracts and establishing the interface between the lipid-matrix and the O-Ext during the production of the O-NLCs [26]. The FTIR evaluations of O-Ext, O-NLCs, and B-NLCs are provided in Figure 6A. At 1050 (S=O stretch), 1125 (C-N stretch), 1425 (C-H bend), 1620 (C=C stretch), 2875 (O-H stretch), 2910 (C-H stretch), and 3400 cm^−1^ (O-H stretch); the B-NLCs bandwidth spiked as reported previously [14]. The FTIR spectrum of O-Ext showed peaks at 800 (C-H »«/bending), 1000 (CO-O-CO «»/stretching), 1600 (O-H »«), and 3300 (C-H «»). The FTIR spectrum of O-NLCs (O-Ext loaded NLCs) revealed characteristic peaks attributed at 700, 1050 (CO-O-CO «»), 1125 (C-N «»), 1425 (C-H »«), 1450 (O-H »«), 1620 (N-H »«), 1730 (C=O «»), 2830 (O-H «»), 2910 (C-H «») cm−1, and 3400 (O-H «»). The O-NLCs spectrum confirmed O-Ext encapsulation in O-NLCs. Similar results were also observed for turmeric loaded into the lipid-matrix [26].

#### 3.2.5. Morphology, Safety, and Permeation of NLCs

The external geomorphology of O-NLCs is given way in Figure 6B. The O-NLCs construction was found via TEM. The figures of O-NLCs were portrayed as rounded-shaped. The mean PS of O-NLCs was ascertained to be 101.2 ± 2.13 nm (Figure 6B). This image confirmed the nano size of O-NLCs holding O-Ext attained by DLS [26]. NLCs are lipids-based nano-formulations that can efficiently deliver dermal, transdermal, and vesicular drugs. The particle size and surface morphology are important physical characters that determine the rate of delivery. Small particles and spherical particles can penetrate the skin barriers easily, where the NLCs, having diameter around 100 nm, can diffuse through the hair follicles and penetrate the skin. A promising effect of using NLCs is the ability to improve skin hydration by creating a protective film in the SC and preventing water loss through the skin [2].

The HaCaT cells viability dealt for 48.0 h, through a series of O-NLCs and O-Ext concentrations (0–250 µg/mL) via MTT assay, given in Figure 6C and ascertained to be concentration-independent. The statistical analysis showed significant (*p* *) changes. These outcomes authorize the O-NLCs as a safe carriers of O-Ext.

The monolayer construction of HaCaT presented the similar absorption possessions such as dermis [49] and was suitable for diffusion experiments, later 17 to 21 days incubation. The mass transport of O-Ext was evaluated across HaCaT after different intervals, i.e., 1, 2, 3, and 6 h, after stabilization of cells monolayer, as shown in Figure 6D. The diffusion of O-Ext was considerably (*p* **) lower than O-NLCs. The outcomes demonstrated that the permeation of O-NLCs was more than that of O-Ext. This considerable upsurge in permeation reveals that O-NLCs are more efficient for O-Ext permeation improvement in topical formulations.

### 3.3. Formulation of O-NLC-E

**Figure 7:**
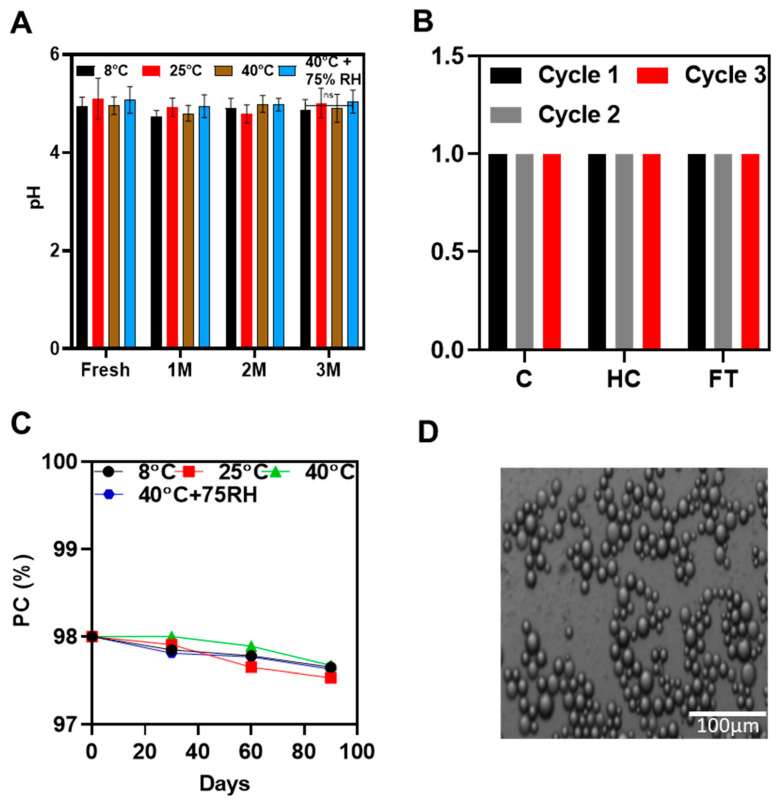
pH studies at different temperatures conditions (**A**), accelerated stability studies (**B**), phenolic contents stability studies (**C**), and microscopic studies (**D**) of O-NLC-E. Data are presented as mean ± SD. n = 3.

A dilution test was conducted by the addition of distilled water in O-NLC-E with stirring; the emulsion was diluted successfully, which shows that the prepared emulsion was O/W. The microscopic image of the diluted emulsion shows the spherical shape and homogeneity of the emulsion (Figure 7D).

#### 3.3.1. Stability Tests

- Accelerated Thermodynamic Stability Studies

Accelerated stability studies were successfully carried out by centrifugation, heating cooling, and freeze–thaw cycles, indicating that formulation could stand the thermal, as well as mechanical, stresses.

##### pH of O-NLCs-E at Different Storage Conditions

pH is a quantifiable measure of the basicity or acidity of topical formulations. The pH variation can cause skin irritation [69]. The pH ranged from 4.8 to 5.3 (Figure 6A) and did not noticeably change during the first, second, and third months of storage under various settings. The O-NLCs-E buffer-capacity sustained the pH invariant. pH changes (PC) were measured at different storage conditions at different time intervals shown in Figure 6B,C; the changes in PC were nonsignificant, calculated by applying two-way ANOVA with post hoc Tukey test. PC of freshly prepared formulation were kept as a standard for comparison. The results show that O-NLC-E demonstrates good buffer capacity during the mentioned shelf life [38].

### 3.4. Ex Vivo Analysis

#### 3.4.1. Ex Vivo Diffusion and Safety Evaluation

**Figure 8:**
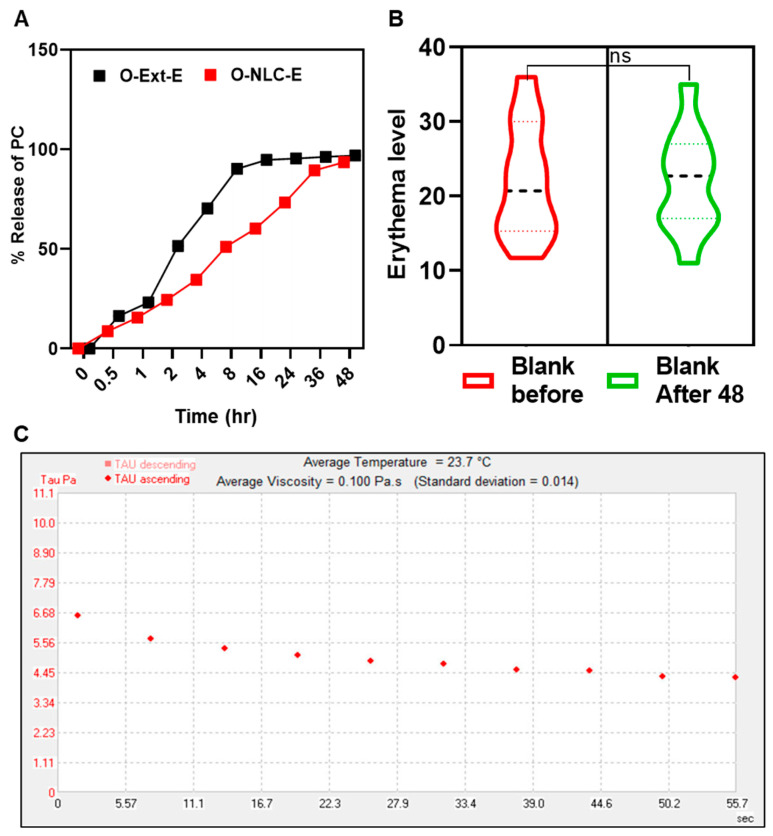
*PC* release studies of O-NLC-E (**A**), safety studies of blank formulations (**B**), and rheological studies of O-NLC-E (**C**). where ns = no significance.

The ex vivo releases percentage was obtained via modified-Franz-diffusion utilizing rat skin as a membrane; results are given in Figure 8A. O-Ext-E release was over 80 of TPC in 8 h, while O-NLCs-E issued 50% of PC gradually. O-NLC-E displayed a slow-release of PC compared to O-Ext-E Figure 8A.

The release data of O-Ext from O-NLC-E and O-Ext-E were tailored by release-kinetic equations. The release with n ≤ 0.43 is governed via the Fickian-diffusion, n ≥ 0.85 by dissolution, and 0.43 < n < 0.8585 via a combination of both [70]. In this study, the Higuchi model (R^2^ = 0.9764) was suitable equation for O-NLC-E. This behavior is premised on the following assumptions: (a) the extract solubility is lesser than the matrix’s initial concentration; (b) there is only one-dimensional diffusion of biomaterials; (c) the system-thickness exceeds bioactive particles in size; (d) there is very little matrix swelling or disintegration; (e) the bioactive component has constant (continuous) diffusivity; and (f) the release system consistently achieves entirely sinking conditions [71]. Patch tests of volunteers for both B-NLC-E, and O-NLC-E was performed (Figure 8B). The results of the safety tests were evaluated via instrument and found safe.

#### 3.4.2. Rheological Behavior of O-NLCs-E

The most popular manifestation of topical formulations is viscosity which effects the incorporated actives to penetrate the skin, along with skin feel and spreadability [72]. Rheograms in Figure 8C showed viscosity of 0.100 Pa.s with 0.014 std, presenting shear-thinning behavior reflecting pseudo-plastic-tendency. Rheology studies the materials flow and deformation under stress [42], and the suitable pseudo-plastic flow enables the thin coating formation that covers the skin [35]. The presented rheological behavior shows smooth-flow afterward tension which leads to create a thin film on the surface of skin.

### 3.5. Antiaging Efficacy of O-NLC-E

#### 3.5.1. Melanin and Erythema

Tyrosinase is an enzyme responsible for the assembly of melanin in the skin layers. Skin melanin gives coloring effects of white to dark skin which is regulated by tyrosinase.

**Figure 9:**
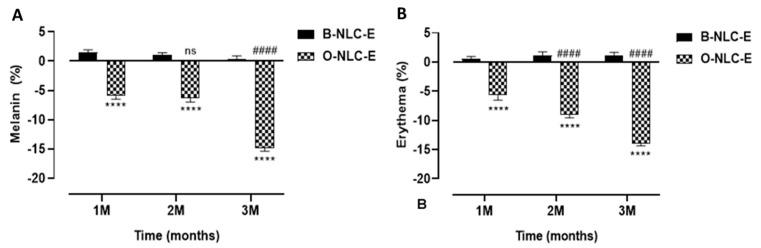
Melanin contents after 1st, 2nd, and 3rd month (**A**); erythema level after 1st, 2nd, and 3rd month (**B**) after application of O-NLC-E. Percent change in skin melanin and erythema after 1st month (1 M), 2nd month (2 M), and 3rd month (3 M) by applying O-NLC-E, along with their respective B-NLC-E. # shows significance difference between O-NLC-E readings taken at different time intervals (1 M: standard) and * shows significance difference between O-NLC-E and their respective B-NLC-E. #### and **** indicates *p* < 0.0001, and ns means no significance. n = 11.

In the case of B-NLC-E and O-NLC-E, it was observed that there were slight variations in skin melanin on the B-NLC-E-treated side, while there was a gradual decrease in melanin values of ONLC-E treated side-up to the 3rd M (Figure 9). An ANOVA test showed that effects produced by O-NLC-E were significant and effects produced by B-NLC-E were insignificant, with respect to time. Through Tukey’s multiple comparisons test, it was ascertained that the melanin of B-NLC-E-treated side at baseline visit vs. 1 M, 2 M, and 3 M were found to be insignificant. Skin Melanin of the O-NLC-E-treated side at baseline visit vs. 1 M and 2 M was insignificant, while baseline visit vs. 3 M was significant. Hesperidin, naringin, eriocitrin, poly-methoxylated flavones (PMF), O-glycosylated flavanone, nobiletin, gallic acid, ferulic acid, and para-coumaric acid 170–174 are found in peel extract. Nobiletin, naringin, and neo-hesperidin were a few of the flavonoids that were discovered as tyrosinase inhibitors, but their inhibiting power was shown to be inferior to kojic acid against mushroom tyrosinase.

Human skin is continuously exposed to environmental stressors such as solar radiations, oxides of nitrogen, ozone, and transition metal ions, as well as some other factors. Oxidative stress with subsequent oxidative damage is more likely to disrupt the normal tocotrienols function in the skin. This skin damage due to the UV is known as skin erythema.

In the case of B-NLC-E and O-NLC-E, there was a minor increase in erythema of B-NLC-E-treated cheek-side, while there was a steady decrease in O-NLC-E treated side-up to the 3rd month (3 M). With the aid of an ANOVA test on effects in erythema values, O-NLC-E produced significant results whilst B-NLC-E produced insignificant values, with respect to time. By applying Tukey’s multiple comparisons test, it was found that skin erythema of BNLC-E-treated side at baseline visit vs. 1 M, 2 M, and 3 M were found to be insignificant, and skin erythema of O-NLC-E-treated side showed a significant decrease compared to baseline vs. 2 M and 3 M. The decline in erythema level by O-NLC-E provided the evidence that it is attributed to gallic acid and hesperidin via downregulation of inflammatory-cascades, MMP1, and oxidative stress.

#### 3.5.2. TEWL and Moisture

**Figure 10:**
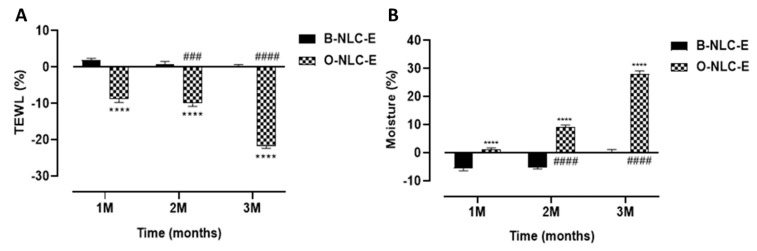
TEWL contents after 1st, 2nd, and 3rd month (**A**); moisture level after 1st, 2nd, and 3rd month (**B**) after application of O-NLC-E. Percent change in skin TEWL and moisture after 1st month (1 M), 2nd month (2 M), and 3rd month (3 M) by applying O-NLC-E, along with their respective B-NLC-E. # shows significance difference between O-NLC-E readings taken at different time intervals (1 M: standard) and * shows significance difference between O-NLC-E and their respective B-NLC-E. #### and **** indicates *p* < 0.0001, ### indicates *p* < 0.001. n = 11.

A correlation between stratum-corneum (SC) hydration and TEWL values exist; both are involved in skin water capacities. The combined skin investigation of trans-epidermal water loss and hydration is appropriate to assess skin responsiveness as shown in Figure 10. In addition, seeing the way that skin interacts with the climate reveals a dire role in the protection of the skin against microbes and increased loss. In the case of B-NLC-E, it was found that there was a minor decrease in TEWL values, while a gradual and prominent increase in TEWL values was reported for O-NLC-E up to the 3rd M. An ANOVA test showed that the O-NLC-E effect was more significant compared to B-NLC-E. The hydration level of O-NLC-E was significantly increased compared to B-NLC-E. In both results, the difference between blank and loaded was due to the bioactive compounds enriched O-Ext.

#### 3.5.3. Sebum and Elasticity

**Figure 11:**
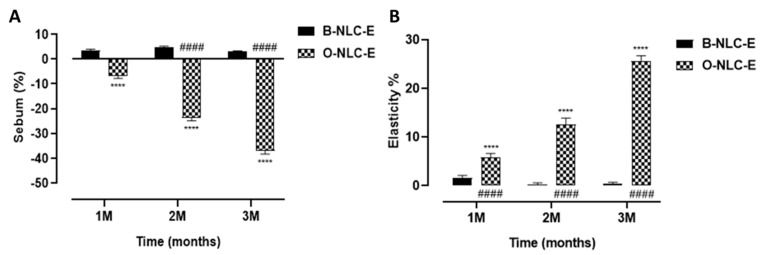
Sebum contents after 1st, 2nd, and 3rd month (**A**); elasticity level after 1st, 2nd, and 3rd month (**B**) after application of O-NLC-E. Percent change in skin sebum and elasticity after 1st month (1 M), 2nd month (2 M), and 3rd month (3 M) by ap-plying O-NLC-E, along with their respective B-NLC-E. # shows significance difference between O-NLC-E readings taken at different time intervals (1M: standard) and * shows significance difference between O-NLC-E and their respective B-NLC-E. #### and **** indicates *p* < 0.0001, n = 11.

The skin sebum is produced by sebaceous glands and harmonized by hormones of the adrenal cortex (corticosteroids), sex hormones (androgens and estrogens), and others. Moreover, 5-α-reductase and androgens receptors are responsible for converting testosterone into dihydrotestosterone (the most potent type enhancing sebum secretion). An increase in the sebum secretion in humans is due to: (1) A testosterone metabolite, dihydrotestosterone (DHT), is produced by 5-α-reductase type-I (2) progesterone, 5α-reductase inhibitor. These hormones upsurge skin sebum by motivating the division of sebocytes. In case of sebum, the value was increased for B-NLC-E, while O-NLC-E decreased the sebum level (Figure 11A), as shown by applying statistical analysis. The decrease in skin sebum is related to inhibit 5α-reductase in the O-NLC-E due to bioactive compounds alpha glucosyl hesperidin, gallic acid, vitamin A, and vitamin C, which can sustain the natural equilibrium of skin oil secretion and enhances the elasticity of the skin by gripping additional oils and eliminating deceased skin cells.

Elastase, on the other hand, is an MMPs enzyme that degrades the elastin fibers. Although elastin fiber can withstand various proteolytic degradations, prolonged exposure to elastase initiates the deterioration of elastin fiber, which causes the skin to lose its integrity, and wrinkle formation initiates. In the case of elasticity measurements, O-NLC-E increases elasticity, while in B-NLC-E, slight reduction occurred (Figure 11B). An increase in elasticity was due to the bioactive compounds of O-Ext which act via downregulation of MMP1. Elastase is responsible for elastin and collagen deprivation, and stimulated via MMP1 and 2.

#### 3.5.4. Panel Test of O-NLCs-E

**Figure 12:**
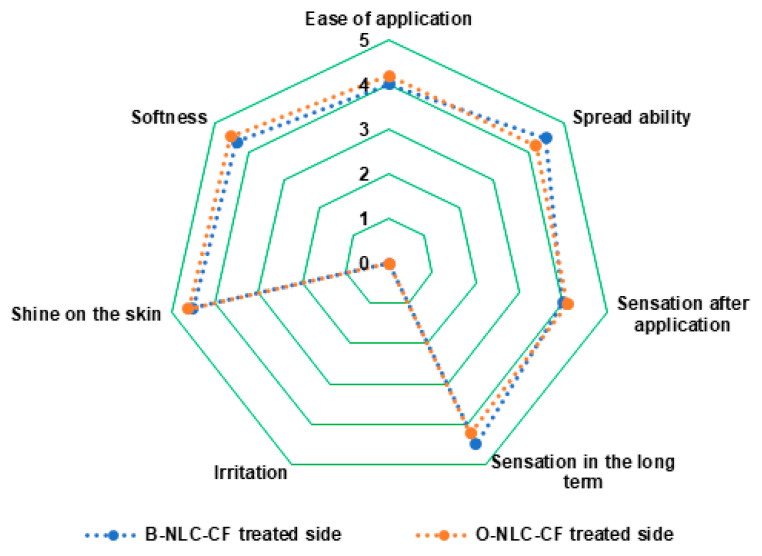
Panel for ease of application, spreadability, sensation just after application, sensation after long-term sense, irritation, shine on the skin, and softness.

To analyze the effectiveness of O-NLC-E and their respective B-NLC-E, a questionnaire was prepared. B-NLC-E received average points for questions relating to the application, spreadability, sensation just after application, long-term sense, irritation, shine on the skin, and softness, represented in Figure 12. We applied a paired sample t-test and found a negligible pairing difference between the average points of sensorial questioners regarding formulations O-NLC-E, and their respective base (B-NLC-E) at different time intervals. Therefore, we concluded that there were no huge changes between formulations O-NLC-E and their respective B-NLC-E regarding the sensory evaluation. Loaded formulations and their respective bases demonstrate similar performance after sensorial analysis. However, silica used as a sensory modifier with surfactants in formulations has played well in addition to sensory attributes of formulations [73]. It is reported from the analysis that formulations are admissible and suitable for topical use.

## 4. Conclusions

The aim of the study was to evaluate orange peels extract for antiaging activity and heavy metals concentration (safety). For this purpose, the extract was loaded into NLCs based on green surfactants (rhamnolipids) and characterized before loading into a secondary formulation (topical O/W emulsion). The results showed good safety profile and high antiaging efficacy. This sustainable approach can mitigate the effects on the skin of the application of substantial bioactive compounds, which successfully overcome the penetration barriers and improve the antioxidant activities against oxidative stresses. This formulation will improve the economic and social compliance, and the topical treatment will fend off oxidative skin problems and early skin aging with ease. In short, the sustainable approach may lessen the skin related social anxiety.

## Figures and Tables

**Table 1:** Concentration of different metals (As, Cr, Co, Cd, Pb, and Fe) in O-Ext.

S. No	Element	Symbols	Concentration Found (ppm)
1	Arsenic	As	0.000
2	Chromium	Cr	0.033 ± 0.001
3	Cobalt	Co	0.083 ± 0.0012
4	Cadmium	Cd	0.028 ± 0.0043
5	Lead	Pb	0.897 ± 0.011
6	Iron	Fe	0.332 ± 0.023

## Data Availability

Data are contained within the article.

## Supplementary Materials

The following supporting information can be downloaded at: https://www.mdpi.com/article/10.3390/bioengineering10070798/s1, Figure S1: Selection of Surfactant, Liquid and Solid-lipids and their Compatibility for NLCs Preparation; Figure S2: Written Consent Sample; Table S1: Bioactive compounds found in GC-MS analysis by Nist library in O-Ext; Table S2: Composition of independent factors in formulations produced via box-benken model; Table S3: Suggested independent and dependent factors by BB model.
