# Supplementary material for: Eco-Friendly Sustainable Nanocarriers to Treat Oxidative Stresses and Skin Aging-Related Ailments, Valorization of a By-Product
Source: Bioengineering (Basel). 2023 Jul 3;10(7):798. doi: 10.3390/bioengineering10070798 (PMC10376035; doi:10.3390/bioengineering10070798)
Supplement: Supplementary file 1 [file bioengineering-10-00798-s001.zip › bioengineering-2435573-supplementary.pdf]

Supporting Information's

Eco-Friendly Sustainable Nanocarriers to Treat Oxidative Stresses and Skin Aging-related Ailments, Valorization of a By-product

Zaheer Ullah Khan[1], Taous Khan[1], Hira Khan[2], Naveed Ullah Khan[3], Yang Ding[4], Atif Ali[1*], and Jiang Ni[5*]

[1] Department of Pharmacy, COMSATS University Islamabad, Abbottabad Campus, Abbottabad, Pakistan; zaheerkhan2092@gmail.com (Z.U.K), taouskhan@cuiatd.edu.pk (T.K), atifali@cuiatd.edu.pk (A.A)

[2] Department of Pharmacy, Abbottabad University of Science and Technology, Abbottabad, Pakistan; hirakhan@aust.edu.pk (H.K)

[3] Department of Pharmacy, CECOS University of Engineering and Emerging Sciences, Peshawar, Pakistan; naveedkhan1676@hotmail.com (N.U.K)

[4] College of Pharmacy, Pharmaceutical Series, China Pharmaceutical University, Nanjing 210000, China; dydszyzf@163.com

[5] Department of Pharmacy, Affiliated Hospital of Jiangnan University, Wuxi 214000, P.R. nj1876348@suda.edu.cn (J.N)

* Correspondence: atifali@cuiatd.edu.pk (A.A), Tel +92-0992-383591-6; nj1876348@suda.edu.cn (J.N), Tel +8613511646862

Supporting Table S1. Bioactive compounds found in GC-MS analysis by Nist library in O-Ext

Hit.	Rev.	For.	Compound Name	M.W	M.F.	CAS. / Lib; Nist.
1	810	646	Dodecanoic acid	200	$C_{12}H_{24}O_2$	143-07-7
2	762	681	Linalool	154	$C_{10}H_{18}O$	78-70-6
3	739	535	Alpha.-terpineol	154	$C_{10}H_{18}O$	98-55-5
4	862	691	Hentriacontane	436	$C_{31}H_{64}$	630-04-6
5	838	435	Cyclohexasiloxane, dodecamethyl	444	$C_{12}H_{36}O_6Si_6$	540-97-
6	658	470	Heptadecane, 1-bromo	318	$C_{17}H_{35}Br$	3508-00-7
9	666	424	L-norvaline, n-(2-methoxyethoxycarbonyl)	443	$C_{25}H_{49}O_5N$	900328-64-7
10	738	473	2,4-di-tert-butylphenol	206	$C_{14}H_{22}O$	96-76-4
11	801	611	2,6-octadien-1-ol, 3,7-dimethyl-, propan	210	$C_{13}H_{22}O_2$	105-91-9
12	702	594	5,5-dimethyl-cyclohex-3-en-1-ol	126	$C_8H_{14}O$	82299-68-1
13	812	710	Benzoic acid, 2-ethylhexyl ester	234	$C_{15}H_{22}O_2$	5444-75-7
14	593	370	Methyl 2-hydroxy-pentacosanoate	412	$C_{26}H_{52}O_3$	118745-42-9
15	873	747	Methyl 11-methyl-dodecanoate	228	$C_{14}H_{28}O_2$	900336-45-1
16	909	634	Tetradecanoic acid, 10,13-dimethyl-, met	270	$C_{17}H_{34}O_2$	267650-23-7
17	765	640	Ethyl 14-methyl-hexadecanoate	298	$C_{19}H_{38}O_2$	900336-64-7
18	782	715	N-hexadecanoic acid	256	$C_{16}H_{32}O_2$	57-10-3
19	795	601	Oleyl oleate	532	$C_{36}H_{68}O_2$	3687-45-4
20	853	718	Isopropyl linoleate	322	$C_{21}H_{38}O_2$	22882-95-7
21	785	554	Hentriacontane	436	$C_{31}H_{64}$	630-04-6
22	721	561	2,6,11-dodecatrienal, 2,6-dimethyl-10-me	218	$C_{15}H_{22}O$	60066-88-8

Supporting Figure S1. Selection of Surfactant, Liquid and Solid-lipids and their Compatibility for NLCs Preparation

**Optimization of NLCs**	**Design of Experiment**	**Model**	**Independent factors**					
	**Stat ease**	**Box-Benken model**	Amount of solid lipids (%)		Amount of lipid mixture (mg)		Amount of surfactant (mg)	
			Upper limit	Lower limit	Upper limit	Lower limit	Upper limit	Lower limit
			99.9	70	600	400	800	400
			**Dependent factors**					
			Size (nm)		Zetapotential (mV)		PDI	

Supporting Table S2. Composition of independent factors in formulations produced via box-benken model

	Dependent factor	Dependent factor	Dependent factor	Size (nm)	ZP (mV)	PDI
Run	**A:lipids (mg)**	**B:SL (%)**	**C:Surfactants (%)**			
F1	500	80	0.5	240	-25	0.8
F2	500	80	1.5	250	-51	0.6
F3	600	89.95	1	164.5	-77.7	0.15
F4	600	99.9	1	368	-59	0.78
F5	600	89.95	1.5	169.6	-78.1	0.19
F6	400	99.9	1	278	-35	0.56
F7	500	99.9	0.5	210	-37.7	0.75
F8	500	89.95	1	160	-76.8	0.23
F9	600	80	1	195	-47.2	0.27
F10	500	89.95	1	157.5	-78.3	0.16
F11	500	99.9	1.5	283	-51	0.67
F12	400	80	1	190	-67	0.16
F13	400	89.95	1.5	203	-71	0.31
F14	400	89.95	0.5	230	-39	0.63
F15	500	89.95	1	201	-75	0.21

Supporting Table S3. Suggested independent and dependent factors by BB model

	Size (nm)	ZP (mV)	PDI	Desirability
**Predicted values**	183.85 ± 15.44	-78.0 ± 3.34	0.18 ± 0.06	0.97
**Actual results**	164.5	−77.7 ± 5.57	0.15±	**Actual**

1.1.1 Statistical analysis of selected models

1.1.1.1 Dependent factor size (nm)

The model is suggested to be significant by the model's F-value of 7.8 and only 0.68% of noise possible. P-values less than 0.05 indicates that model terms are significant. In this case B, B^2 are significant model terms. The Lack of Fit F-value of 3.16 indicates that Lack of Fit is not significant relative to the pure error and non-significant lack of fit is considered good as the model should to fit.

1.1.1.2 Dependent factor ZP (mV)

The Model F-value of 19.81 shows that model is significant with only a 0.02% chance of noise. On the basis of P-value (< 0.05) the model terms are significant. In this case C, AB, B^2, C^2 are significant model terms. The Lack of Fit F-value of 17.77 implies there is a 5.42% chance that a Lack of Fit F-value this large could occur due to noise.

1.1.1.3 Dependent factor PDI

The Model F-value of 11.07 suggests that model is significant with only a 0.11% chance of noise. P-values less than 0.05 indicate model terms are significant. In this case B, C, B^2, C^2 are significant model terms. The Lack of Fit F-value of 15.96 implies there is a 6.03% chance that a Lack of Fit F-value this large could occur due to noise.

Supplementary Figure S2. Written consent sample

Written consent sample

COMSATS UNIVERSITY ISLAMABAD, ABBOTTABAD CAMPUS
CONSENT TO PARTICIPATE IN RESEARCH STUDY
Short Form Written Consent (used with oral consent form)

STUDY TITLE: "Complete Skin Investigation of -*Citrus Sinensus* Extract Loaded Nano-lipid Carriers Sustainable Topical Formulation" on skin of volunteers in Population of Abbottabad; Valorization of a by-product

Principle Investigator: Dr. Atif Ali

Investigator: Zaheer Ullah Khan

WRTTIEN CONCENT

I confirm that the researcher has explained the elements of informed consent to the participant. The subject knows that their participation is voluntary, and that they do not need to answer all questions. The purpose of the research as well as the risks and benefits have been explained. The procedures as well as the time commitment have been outlined. The participant understands issues of confidentiality.

Participant name ________________________

Participant signature__________________________
